# Molecular mechanism of biocompatible clusteroluminogens from citric acid and l-lysine

**DOI:** 10.1039/d5ra09240j

**Published:** 2026-01-13

**Authors:** Qiannan Zhang, Pingchuan Sun, Baohui Li

**Affiliations:** a School of Physics, Nankai University Tianjin 300071 P. R. China baohui@nankai.edu.cn; b Key Laboratory of Functional Polymer Materials of Ministry of Education and College of Chemistry, Nankai University Tianjin 300071 P. R. China spclbh@nankai.edu.cn

## Abstract

Clusteroluminescence in non-conjugated systems has garnered significant attention for the development of advanced light-emitting materials, however, the understanding of the underlying mechanism remains a challenge. Herein, we report a facile, one-step strategy to prepare unconventional dual-mode luminescent materials by thermal treatment of aqueous citric acid (CA) and l-lysine (Lys). These materials exhibit bright fluorescence (The quantum yield is up to 43.2%) and remarkably long-lived room-temperature phosphorescence (RTP, up to 5 s). Combined experimental characterization and theoretical calculations were used to reveal the underlying dual emission mechanisms. Theoretical calculations revealed a reduced HOMO–LUMO energy gap upon blending of the CA and Lys and formation of ionic interaction in CA and Lys mixtures. Blue IRI isosurface calculation demonstrates the formation of H⋯O and H⋯N intermolecular weak interactions, which promote efficient electron transitions, enhancing molecular excitability. This structural characteristic increases the probability of radiative decay to the ground state, thereby improving long-wavelength fluorescence efficiency. The observed trends in fluorescence and phosphorescence spectra were in excellent agreement with theoretical calculation results, providing further mechanistic insights into the luminescence behavior. This work provides a facile strategy for the preparation of dual-mode luminescent materials and new insight into understanding the molecular mechanism of clusteroluminescence in non-conjugated systems.

## Abstract 2

One-step synthesis of dual-mode luminescent materials using CA/Lys gives high fluorescence (43.2%) and ultralong RTP (5 s). Study shows interactions/ionic bonds boost electron transitions, explaining clusteroluminescence in non-conjugated systems.

## Graphical abstract

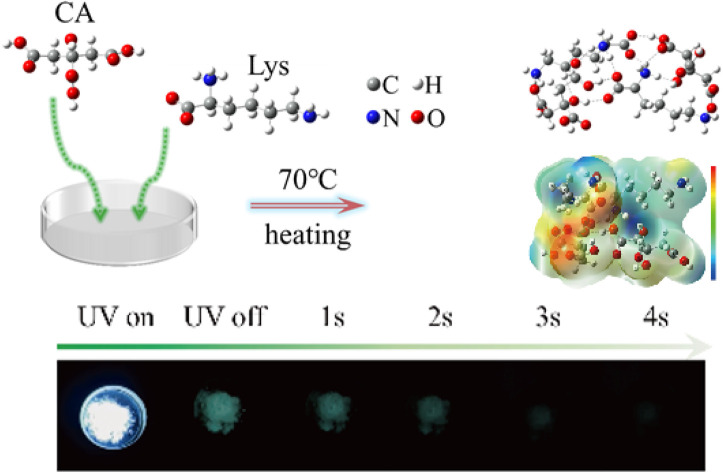

## 1. Introduction

Intrinsic fluorescence from non-conjugated polymers and biomacromolecules in their clustered states – termed cluster-triggered emission (CTE) – has emerged as a significant phenomenon.^1–4^ This behaviour is observed in diverse systems, including synthetic polymers^5–7^ like polyimides,^8^ maleimide,^9^ maleic anhydride/vinyl acetate alternating copolymers (PMV),^10^ polyethylene glycol,^11,12^ and polyesters^13–15^ as well as natural biopolymers such as rice constituents (starch and cellulose),^16,17^ structural proteins,^18–20^ and mono-, di-, oligo-polysaccharides.^21–23^ Compared to conventional aromatic luminescent materials, clusteroluminogen materials synthesized from renewable components offer significant advantages.^24–27^ These materials possess superior biocompatibility and biodegradability, thus avoiding the potential cytotoxicity and long-term accumulation risks associated with aromatic rings and enhancing their suitability for biological applications.^28–30^ Furthermore, their enhanced sustainability stems from all components being derived from renewable natural sources, making them environmentally friendly and biodegradable.

However, current strategies for tuning CTE emission typically require complex chemical synthesis, limiting simplicity and scalability,^31–34^ especially for non-conjugated systems with both fluorescence and room-temperature phosphorescence (RTP) properties.^35–40^ Furthermore, a fundamental understanding of the photophysical mechanisms driving CTE, particularly concerning electronic interactions within the cluster environment, the factors governing efficiency, and the potential for dual emission (fluorescence and phosphorescence), remains largely elusive.^41–45^ Elucidating these mechanisms is crucial not only for advancing fundamental knowledge of light–matter interactions in non-traditional systems but also for the rational design of next-generation, tuneable luminescent materials with tailored properties.^46–48^

**Fig. 1:**
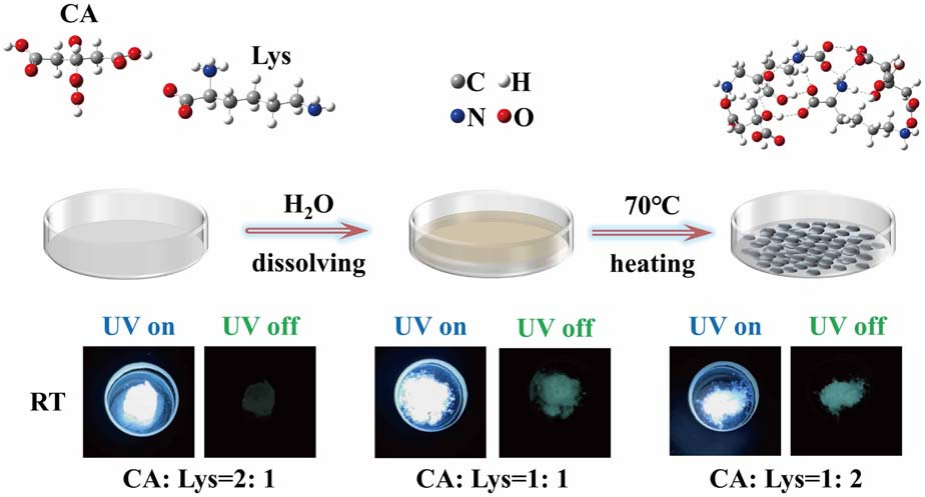
Schematic illustration of the fabrication process for citric acid and l-lysine aggregate samples, along with photographs taken before and after turning off a UV 365 nm light source.

Herein, we present a facile one-step strategy to prepare unconventional dual-mode luminescent materials through mild thermal treatment of aqueous citric acid (CA) and l-lysine (Lys) mixtures at a precisely controlled CA : Lys ratio (Fig. 1). This study aims to elucidate the regulatory mechanisms underlying the dual-mode emission at the microscopic level, including molecular orbital energy levels, electron cloud distribution, and the evolution of weak intermolecular interactions, by integrating systematic experimental characterization with density functional theory (DFT) calculations. The results show that at a specific molar ratio, the molecular interactions between CA and Lys are significantly enhanced, effectively suppressing molecular motion, promoting efficient electron transitions, and increasing molecular excitability, thereby improving radiative transition efficiency. This study not only provides a straightforward approach for preparing dual-mode luminescent materials but also provides new insights into the design of efficient and multifunctional light-emitting systems, while advancing the fundamental understanding of luminescence phenomena in non-conjugated systems by clarifying the microscopic photophysical processes governing their emission behavior.

## 2. Experimental

### 2.1 Materials

Citric acid (CA) and l-lysine (Lys), both with a purity of 99.5%, were purchased from Shanghai Macklin Biochemical Technology Co., Ltd. All solvents were commercially available and used as received.

### 2.2 Synthesis of CA : Lys mixture

Citric acid and l-lysine were employed as starting materials and dissolved in distilled water at molar ratios (CA : Lys) of 2 : 1, 1 : 1, and 1 : 2 (Fig. 1). Three different molar ratio systems were prepared by dissolving citric acid (CA) and l-lysine (Lys) in 10 mL distilled water: (i) 2 : 1 (CA : Lys) with final concentrations of 0.76 mol L^−1^ CA and 0.38 mol L^−1^ Lys; (ii) 1 : 1 (CA : Lys) with 0.59 mol L^−1^ for both components; (iii) 1 : 2 (CA : Lys) with 0.41mol L^−1^ for CA and 0.82 mol L^−1^ Lys. Three fluorescent products were obtained by heating the mixtures at 70 °C in air for 10 h, followed by vacuum drying for 3 h, designated as CA : Lys = 2 : 1, CA : Lys = 1 : 1, and CA : Lys = 1 : 2.

### 2.3 Photoluminescence characterization

Photoluminescence spectrum and solid-state quantum yield measurements were performed using an FS5 spectrophotometer (Edinburgh Instruments Ltd.) with excitation provided by either a xenon lamp or a microsecond flash lamp at selected wavelengths (*λ*_ex_ = 320, 330, 340, 350, and 360 nm). Sample images were captured using a digital camera under UV illumination.

### 2.4 Fourier transform infrared spectroscopy (FT-IR) characterization

FT-IR spectroscopic analysis was conducted using a TENSOR II spectrometer (Bruker, Germany) equipped with an attenuated total reflection (ATR) sampling accessory. Spectra were acquired with 16 cumulative scans at a resolution of 4 cm^−1^ and a scanning frequency of 1.6 kHz, covering the mid-infrared region (450–4000 cm^−1^) to identify characteristic functional group vibrations.

### 2.5 Low-field nuclear magnetic resonance (NMR) measurements

Low-field nuclear magnetic resonance (NMR) measurements were conducted on a Bruker Minispec mq20 spectrometer operating at a proton resonance frequency of 20 MHz. The sample temperature was maintained using a BVT3000 heater within a flowing air-protected environment. To mitigate potential oxidation, all samples were flame-sealed under vacuum prior to analysis. Key instrument parameters included a 90° pulse length of ∼3.7 µs and a dead time of 14.7 µs.

### 2.6 Wide-angle X-ray diffraction (XRD)

Powder XRD patterns were collected on a Rigaku SmartLab multipurpose X-ray diffractometer (Rigaku Corporation, Japan) using Cu Kα radiation (*λ* = 1.5406 Å). Samples were mounted on a zero-background silicon holder and scanned over a 2*θ* range of 0–80° with a step size of 0.02° and a counting time of 1 s per step.

### 2.7 Small-angle X-ray scattering (SAXS)

SAXS measurements were performed using a Xeuss 3.0 system (Xenocs SA, France) equipped with a GeniX 3D microfocus source (Cu Kα, *λ* = 1.5406 Å) and a Pilatus 1 M detector. Samples were loaded in a dedicated sample holder and measured in transmission mode, covering a *q*-range of 0–4 Å^−1^ (corresponding to *d*-spacings from ∞ ∼ 1.5 Å), where *q* = (4π sin *θ*)/*λ*.

## 3. Results and discussion

### 3.1 Microstructure characterization of CA : Lys mixtures

**Fig. 2:**
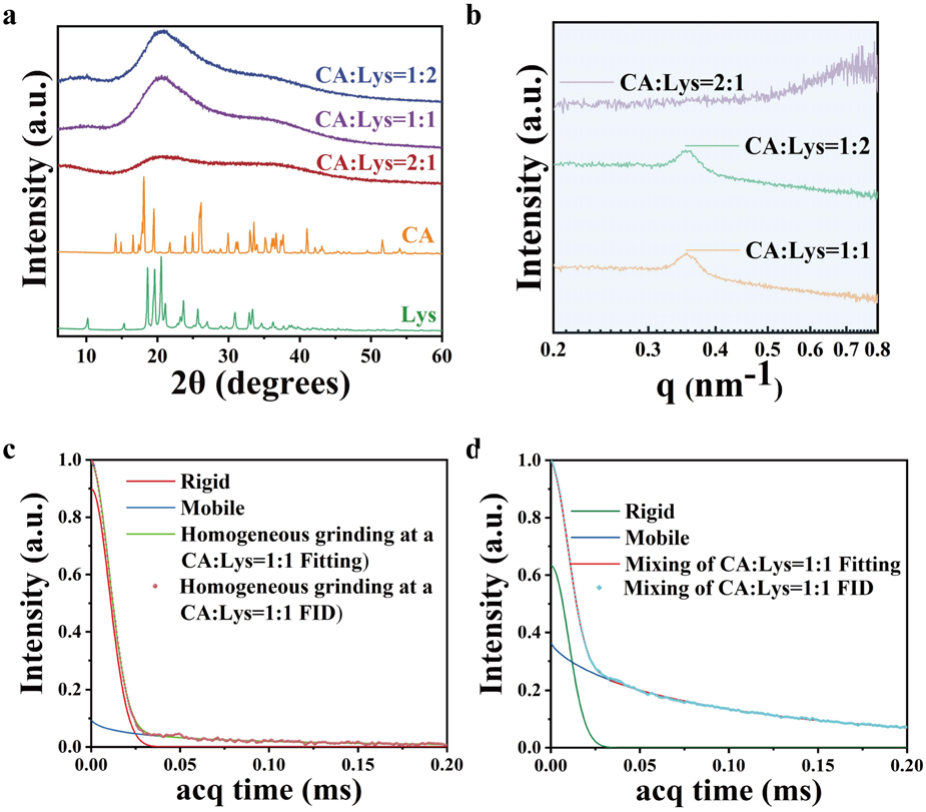
Structural characterization of CA/Lys composites: (a) comparative X-ray diffraction (XRD) analysis of CA : Lys systems with 2 : 1, 1 : 1, and 1 : 2 molar ratios alongside pristine CA and Lys controls; (b) small-angle X-ray scattering (SAXS) patterns of CA : Lys composites at varying stoichiometric ratios (2 : 1, 1 : 1, 1 : 2). (c) FID signal and fitted curve of CA : Lys = 1 : 1 molar ratio grinding measured by MSE experiment. (d) FID signal and fitted curve of CA : Lys = 1 : 1 molar ratio blending measured by MSE experiment.

The 2 : 1 product formed as bulk solids, while the 1 : 1 and 1 : 2 products exhibited granular morphology. The amorphous aggregate of CA and Lys led to salt formation through enhanced ionic interactions^33^ (SI Fig S1). And the chemical structure of the mixtures was characterized using proton nuclear magnetic resonance (^1^H NMR) spectroscopy. As shown in SI Fig S5, the ^1^H NMR spectra of the sample heated at 70 °C and the physically blended sample at room temperature are completely identical. This indicates that under our experimental conditions, no chemical reaction occurred between citric acid and lysine, further ruling out the possibility of carbon dot formation. Both citric acid and lysine are multifunctional molecules, and their blending can lead to the formation of complexes with varying stoichiometries or connectivity, which hinders molecular rearrangement and diffusion during crystallization. Furthermore, the rapid solvent evaporation process likely further impedes the ordered arrangement of molecules. Collectively, these factors prevent the molecules from achieving long-range ordered packing, ultimately resulting in an amorphous structure. This structural modification, characterized by strengthened intermolecular hydrogen bonding networks and the reorganization of the two molecular components, establishes the necessary structural prerequisites for the emergence of fluorescent properties. To investigate the origin of the strong fluorescence and room-temperature phosphorescence observed in these mixtures, we conducted structural characterizations using X-ray diffraction (XRD) and small-angle X-ray scattering (SAXS). As shown in Fig. 2a and b, the XRD results show that upon blending CA with Lys, the distinct diffraction peaks corresponding to both components vanished completely, indicating the disruption of their crystalline lattices. Instead, the mixtures exhibited only broad diffraction features, characteristic of amorphous structures. Notably, SAXS results confirm that the CA : Lys = 2 : 1 mixture maintains complete nanoscale amorphousness. This directly correlates with its experimentally observed non-granular morphology. In contrast, the CA : Lys = 1 : 1 and 1 : 2 mixtures exhibit weak periodic ordering (∼1.77 nm), corresponding to their distinctly observed granular morphologies. The structural evolution suggests ionic cluster formation in these two mixtures, which likely contributes to their luminescent properties.

### 3.2 The low-field nuclear magnetic resonance (LF-NMR) analysis of CA : Lys mixtures prepared by grinding *vs.* mixing

As illustrated in Fig. 2c and d, LF-NMR was employed to analyse blended samples of CA and Lys (1 : 1 molar ratio) prepared *via* two distinct methods: (1) uniform mechanical grinding and (2) dissolution in water followed by solvent evaporation. As shown in SI Table S1, the ground sample exhibited a predominantly rigid phase (95.75%, T_2_Rigid = 0.0147 ms), with only a minor flexible phase contribution (4.25%, T_2_Mobile = 0.1 ms). This suggests that the mechanical grinding process preserved the original crystalline structure of the starting materials, with minimal alterations in intermolecular interactions. The absence of significant changes in T_2_ relaxation times further indicates that no new chemical bonds or substantial non-covalent interactions were formed during the grinding process. In contrast, the solution-processed sample displayed a markedly different phase distribution, with the rigid phase decreasing to 63.31% (T_2_Rigid = 0.0138 ms) and the flexible phase increasing substantially to 36.69% (T_2_Mobile = 0.1 ms) (Fig. 2d and SI Table S1). This result implies that the dissolution and solvent removal process disrupted the native crystalline arrangement, facilitating enhanced intermolecular interactions between CA and Lys. The above results are in good agreement with XRD findings. The increased flexible phase proportion likely arises from the formation of additional hydrogen-bonding networks and/or ionic pairs during solution processing.

### 3.3 Photophysical properties of chromophores in solution

**Fig. 3:**
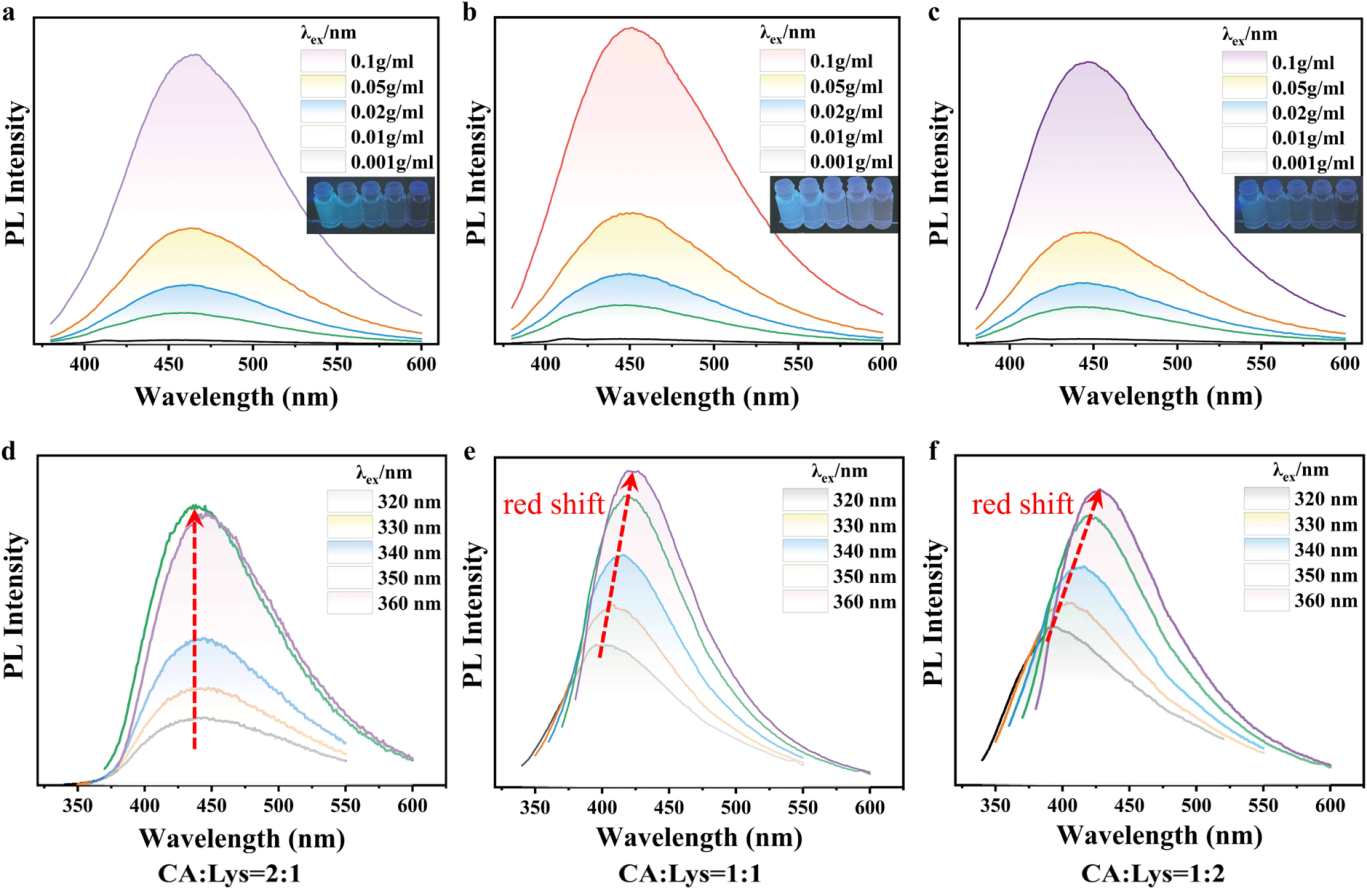
Photoluminescence (PL) spectra of (a–c) aqueous solutions at different concentrations and (d–f) solid samples excited at different wavelengths, with varying CA : Lys molar ratios: (a and d) 2 : 1, (b and e) 1 : 1, and (c and f) 1 : 2.

To investigate the CTE properties of the composite system, samples with citric acid CA to Lys molar ratios of 2 : 1, 1 : 1, and 1 : 2 were dissolved in water to prepare solution systems of varying concentrations. The photoluminescence (PL) behavior of solutions at different concentrations was systematically investigated under 360 nm excitation. As illustrated in Fig. 3a–c, the PL intensity of the three solutions increases with increasing CA–Lys system concentration, indicating distinct aggregation-enhanced emission behaviour. The PL emission peaks (*λ*_em_) for the CA : Lys ratios of 2 : 1, 1 : 1, and 1 : 2 were observed at 466 nm, 451 nm, and 447 nm, respectively.

### 3.4 Photophysical properties of chromophores in the aggregate state

The solid-state PL spectra of solid-state CA : Lys mixtures were systematically measured with three CA : Lys molar ratios (2 : 1, 1 : 1, and 1 : 2) under various excitation wavelengths (Fig. 3d–f). All samples exhibited distinct short-wavelength emission peaks at 447 nm, 423 nm, and 428 nm, respectively. Notably, significant variations in PL intensity were observed upon 360 nm excitation. Quantum yield measurements revealed exceptional values of 43.2%, 37.1%, and 35.4% for the 2 : 1, 1 : 1, and 1 : 2 ratios, respectively. Among these mixtures, the CA : Lys = 2 : 1 system demonstrated particularly intense fluorescence emission. These values are comparatively high among those reported for non-conventional luminescent materials in this emission range.^49–51^ For the bulk blend with a CA : Lys ratio of 2 : 1, the photoluminescence (PL) intensity increases sharply with increasing excitation wavelength (*λ*_ex_), while the emission wavelength (*λ*_em_) remains nearly constant. The well-defined emission peaks are characteristic of the *n*–π* transition associated with C=O groups.^52^ For mixtures with CA : Lys ratio of 1 : 1, a similar rapid enhancement in PL intensity is observed upon increasing *λ*_ex_. However, in this case, *λ*_em_ exhibits a slight but discernible red shift. A more pronounced trend emerges in the CA : Lys = 1 : 2 mixture, where the PL intensity again increases significantly with *λ*_ex_, accompanied by a substantial red shift in *λ*_em_. Comparative analysis of the 1 : 1 and 1 : 2 solid mixtures reveals that the higher Lys content in the 1 : 2 system leads to a more prominent excitation-dependent emission (EDE) effect. These results demonstrate that the excitation wavelength dependence arises from lysine-mediated interactions, predominantly through three synergistic mechanisms: (1) ionic bond formation between charged functional groups, and (2) enhanced inter-molecular hydrogen bonding networks.

### 3.5 Room temperature phosphorescence of CA : Lys mixtures

**Fig. 4:**
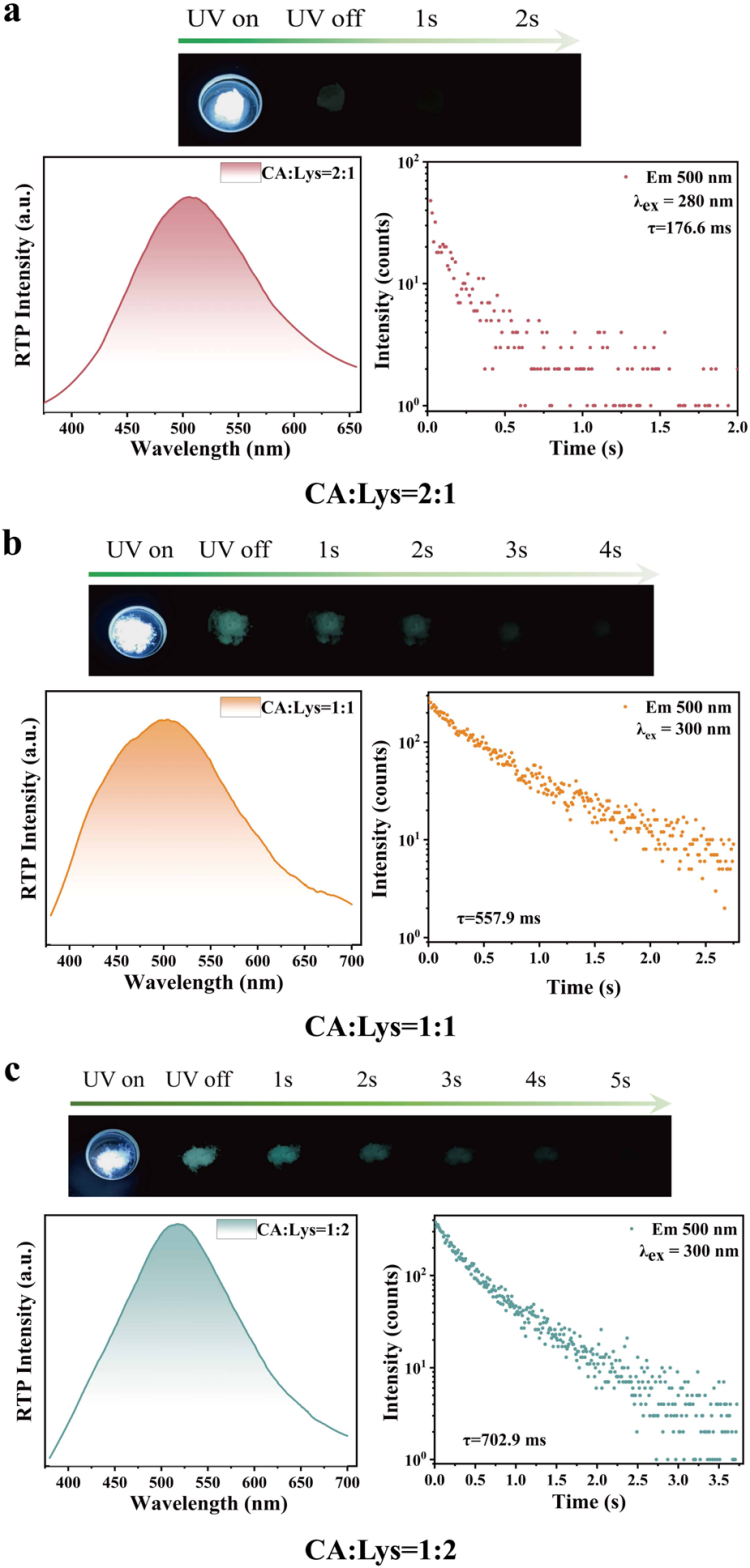
Photophysical characterization of CA : Lys composite systems with varying stoichiometric ratios: (a) 2 : 1, (b) 1 : 1, and (c) 1 : 2 molar compositions. Each panel displays, in the upper section, luminescence photographs taken under UV irradiation and at different duration times after ceasing the UV lamp; the lower section presents corresponding phosphorescence spectral features on the left, and lifetime decay curves on the right.

Furthermore, as shown in Fig. 4, the mixture systems with CA : Lys molar ratios of 2 : 1, 1 : 1, and 1 : 2 exhibit persistent room-temperature phosphorescence (RTP). Compared with previously reported citrate-based luminescent materials, this work demonstrates significant improvements in both synthetic methodology and luminescence performance. Earlier studies generally required extreme conditions—such as sustained heating above 150 °C, high pressure, or microwave assistance—to drive the reactions,^53–56^ aiming to promote complex intermolecular dehydration, polymerization, or carbonization for chromophore formation. In sharp contrast, the present work develops an exceptionally simple and mild room-temperature solution-blending method. Under ambient conditions, these samples demonstrate distinct photoluminescent properties upon 365 nm excitation. For the CA : Lys = 2 : 1 mixture, intense emission was observed under UV irradiation. Upon cessation of excitation, a weak green afterglow persisted for approximately 2 seconds, corresponding to a phosphorescence band with an emission maximum at 506 nm and a lifetime of 176.6 ms. Similarly, the CA : Lys = 1 : 1 and 1 : 2 samples display strong blue luminescence when irradiated at 365 nm. After turning off the UV light, the green afterglow remained visible for ∼4 and 5 seconds, with phosphorescence peaks at 520 and 525 nm, and lifetimes of 557.9 and 702.9 ms, respectively.

### 3.6 TD-DFT calculations and the proposed mechanism of fluorescence emission

**Fig. 5:**
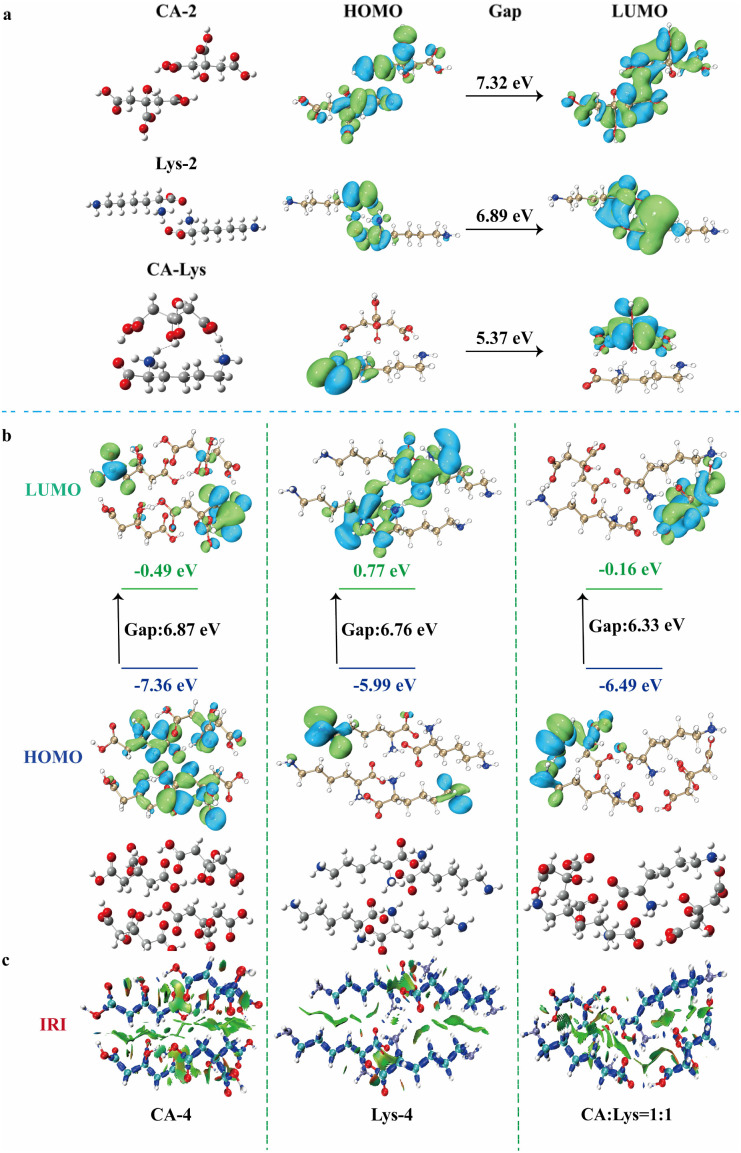
(a) Frontier molecular orbital characteristics of optimized structures: CA dimer, Lys dimer, and CA : Lys mixture system, illustrating HOMO–LUMO distributions and their contributions to fluorescence emission; (b) Frontier molecular orbital characteristics of the CA tetramer, Lys tetramer, and CA–Lys mixture tetramer, highlighting the HOMO and LUMO molecular orbitals responsible for fluorescence; (c) corresponding isosurface representations of the interaction region indicator (IRI) function.

To further elucidate the mechanism underlying the fluorescence and room-temperature phosphorescence emission observed in mixed samples, we conducted systematic theoretical investigations using time-dependent density functional theory (TD-DFT). The calculations demonstrate that the blended dimers exhibit significantly enhanced luminescence efficiency. Structural optimizations were performed at the B3LYP/6-31G* level of theory implemented in the Gaussian 16 and Multiwfn software packages.^57,58^ In contrast, the CA : Lys = 1 : 1 mixture displayed distinct electronic properties. The strengthened intermolecular interactions, particularly H⋯O, H⋯N, and C=O bonds, facilitated orbital redistribution. Notably, the HOMO and LUMO became spatially separated on different molecular units, demonstrating clear charge transfer (CT)features. This electronic reorganization results in a reduced HOMO–LUMO gap (5.37 eV), which attributes to the enhanced long-wavelength fluorescence efficiency (Fig. 5a). The three CA : Lys ratios (2 : 1, 1 : 1, 1 : 2) mixtures reveal progressive changes in electronic structure, with the energy gap (Δ*E*) systematically decreasing (Fig S2). In order to obtain a more accurate aggregated structure, further optimization was performed on the tetrameric systems (CA-4, Lys-4, and CA : Lys = 1 : 1), leading to the determination of these ground-state energy levels in the optimized conformations. As demonstrated in Fig. 5b, the optimized geometries reveal a distinct spatial separation between HOMO and LUMO orbitals across different molecular subunits, indicating significant CT character. Notably, the CA : Lys = 1 : 1 mixtures exhibit enhanced intermolecular connectivity through multiple interaction motifs, including ionic bond, H⋯O, H⋯N and C=O contacts, which collectively establish an extensive electron transfer network (Fig. 5c). This structural configuration leads to a substantial reduction in the HOMO–LUMO energy gap, thereby significantly lowering the energy barrier for electronic transitions.

### 3.7 Analysis of the degree of delocalization of various orbitals in molecules

**Fig. 6:**
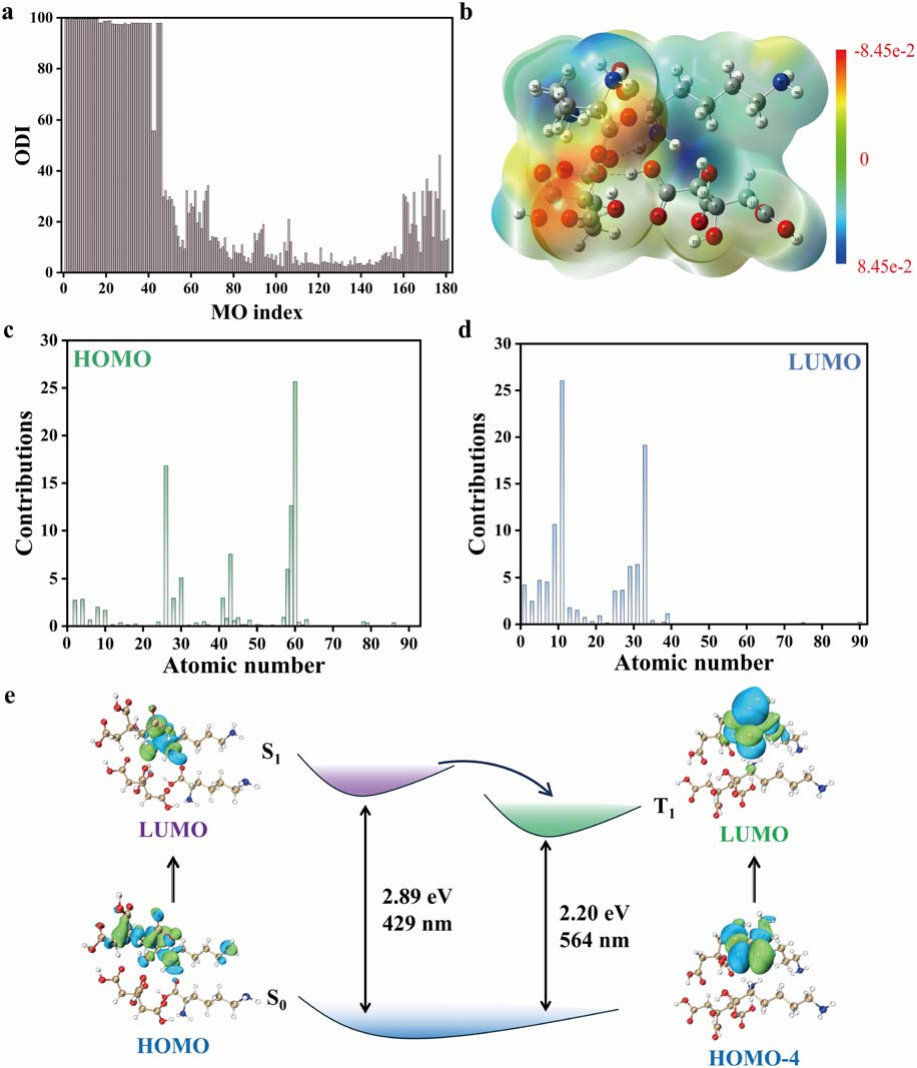
(a) Calculated orbital delocalization indices (ODIs) and corresponding molecular structure of the CA–Lys mixture. (b) Electrostatic potential distribution on the molecular surface of the CA–Lys mixture. (c) Atomic contributions to the ODI of the HOMO NTO and(d) LUMO NTO in CA–Lys. (e) DFT-calculated electronic energy levels of singlet (S_1_) and triplet (T_1_) states for the CA : Lys = 1 : 1 tetrameric mixture.

The degree of orbital delocalization in the blended molecular system (CA : Lys = 1 : 1) was quantitatively assessed using the Orbital Delocalization Index (ODI), where a smaller ODI value indicates greater delocalization. As demonstrated in Fig. 6a, the first 46 orbitals correspond to core electrons, and these exhibit relatively high ODI values (some approaching only ∼50%, attributable to their simultaneous presence in the core regions of two adjacent atoms). Notably, the HOMO and LUMO orbitals exhibit significantly low ODI values, indicative of strong delocalization. This enhanced delocalization facilitates intermolecular electron transfer, thereby playing a critical role in stabilizing the molecular system. As illustrated in Fig. 6c and d, further analysis of orbital contributions reveals that atoms 26, 30, 43, 58, 59, and 60 dominate the HOMO ODI, whereas atoms 11, 29, 31, and 33 contribute predominantly to the LUMO ODI. These observations suggest that upon blending CA with Lys, electronic interactions lead to spatial charge redistribution between the two molecules, mediated by non-covalent orbital overlap. As illustrated in Fig. 6b, the molecular surface electrostatic potential analysis revealed that the CA–Lys mixture maintains an obvious electrostatic interaction. Notably, the CA moiety exhibits a localized electronegative potential, whereas the Lys region displayed a distinct positive potential (Fig. 6b). This electrostatic polarity suggests the migration of H^+^ from CA to Lys, accompanied by concomitant charge transfer, which aligns well with ODI results.

### 3.8 TD-DFT calculations and the proposed mechanism of fluorescence and ultralong phosphorescence

To elucidate the origin of RTP in the CA : Lys mixtures, we performed systematic DFT and TD-DFT calculations on a tetrameric model with a 1 : 1 stoichiometric ratio. The molecular structure of the model was first optimized, and then the corresponding singlet (S_1_) and triplet (T_1_) energy levels were computed. As shown in Fig. 6e, the calculated S_1_ energy (2.89 eV) and T_1_ energy (2.20 eV) correspond to fluorescence and phosphorescence emission wavelengths of 429 nm and 564 nm, respectively, which align well with experimental observations (423 nm and 520 nm). This close agreement of theoretical and experimental results validates the reliability of the tetrameric model. The spatial charge transfer and intermolecular interactions (*e.g.*, hydrogen bonding or ionic bonding) between CA and Lys significantly suppress the vibration, rotation, and collision of phosphorescent molecules, thereby blocking the main pathway of non-radiative decay. Under ambient conditions, oxygen quenching is one of the primary reasons why phosphorescence is seldom observed at room temperature. However, in this system, the aggregation driven by interactions between CA and Lys greatly restricts the diffusion and penetration of oxygen molecules, preventing them from accessing and quenching the T_1_ excitons “locked” inside. This mechanism not only facilitates efficient intersystem crossing (ISC) from S_1_ to T_1_–T_3_ but also markedly extends the phosphorescence lifetime. The presence of multiple ISC channels further enhances the conversion efficiency from singlet to triplet states, thereby ensuring stable and efficient RTP emission in the CA : Lys system.

### 3.9 Application of CA and Lys mixtures

**Fig. 7:**
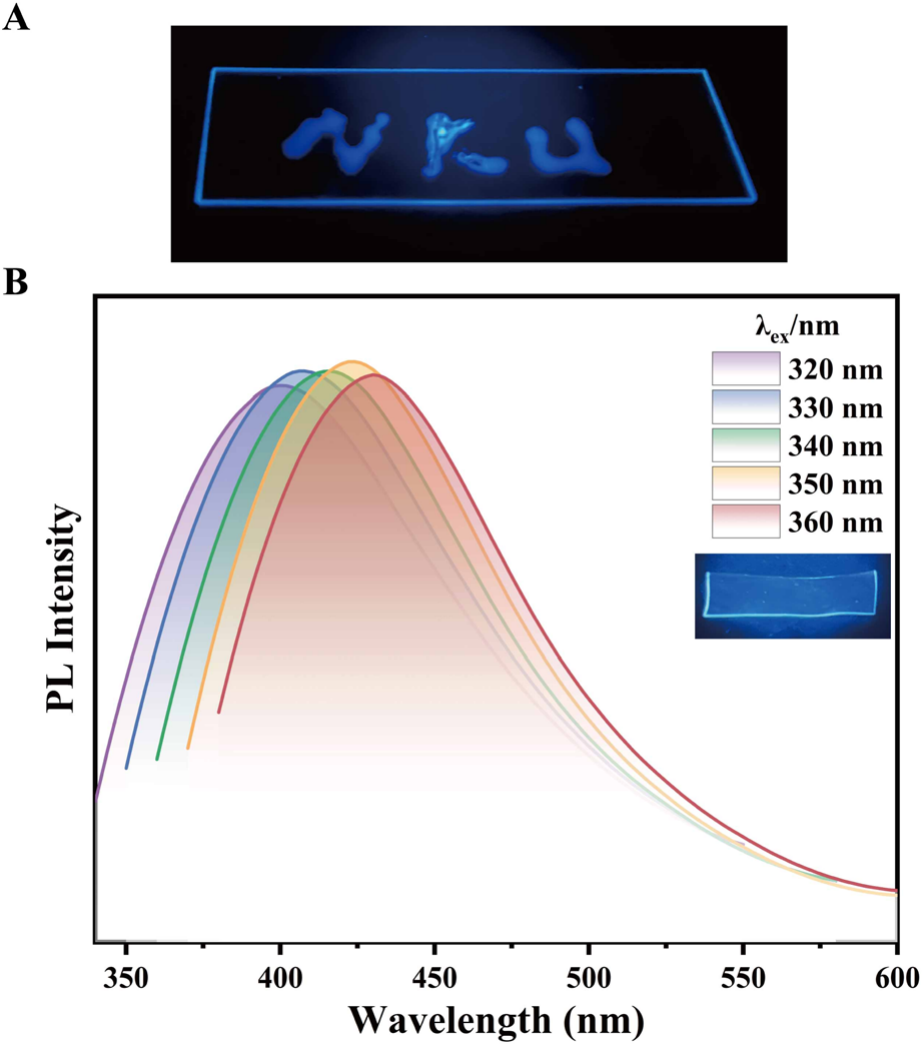
(a) UV-excited (365 nm) fluorescence image of CA–Lys displaying “NKU” patterning on glass substrate. (b) Polarized photoluminescence microscopy image of CA-Lys-embedded PVA composite film.

Moreover, CA–Lys demonstrates strong adhesive properties across diverse substrates. For example, it adheres readily to glass surfaces, enabling facile patterning. As illustrated in Fig. 7a, a glass substrate labelled with the abbreviation “NKU” (denoting “Nankai University”) using CA–Lys is shown alongside its corresponding fluorescence image under 365 nm UV excitation. Leveraging CA–Lys's notable water solubility, biocompatibility, and non-toxicity, we fabricated a composite film by integrating it with polyvinyl alcohol (PVA) (Fig. 7b). The resulting film exhibits high fluorescence intensity and excitation-dependent emission characteristics. Fluorescence spectra of the CA-Lys-embedded PVA composite film from three repeated experiments showed consistent reproducibility and repeatability (Fig. S6).

## 4. Conclusions

In summary, we successfully synthesized highly fluorescent and long-lived room-temperature phosphorescent materials by a simple thermal treatment of aqueous citric acid and l-lysine. It is demonstrated that an efficient amorphous RTP system can be constructed by regulating non-covalent interactions between non-conjugated biomolecules, without the need for conventional chemical bonding. The underlying mechanism involves the disruption of crystallinity in the pristine components and the synergistic effect of dynamic ionic–hydrogen bonds formed between complementary functional groups, such as carboxyl and amino groups. This interaction leads to the formation of a rigid, spatially confined, interconnected molecular network. Theoretical calculations corroborate that the blended material induces pronounced intermolecular and spatial charge-transfer characteristics, effectively narrowing the excited-state energy gap and enhancing spin–orbit coupling. As a result, both intersystem crossing and radiative decay processes are synergistically promoted, providing further mechanistic insights into the luminescence behaviour. This work provides new insights into organic fluorescence and phosphorescence of molecular mixtures, laying a foundation for developing sustainable and customizable phosphorescent materials.

## Author contributions

Qiannan Zhang: conceptualization, data curation, writing original draft, and visualization. Baohui Li: conceptualization, resources, writing – review & editing. Pingchuan Sun: resources, writing – review & editing, supervision, project administration, and funding acquisition.

## Conflicts of interest

There are no conflicts to declare.

## Supplementary Material

RA-016-D5RA09240J-s001

## Data Availability

The data supporting this article have been included as part of the supplementary information (SI). Supplementary information: Tables S1, FT-IR spectra, ^1^H-NMR and theoretical calculation details.ion details. See DOI: https://doi.org/10.1039/d5ra09240j.
