# Supplementary material for: Molecular mechanism of biocompatible clusteroluminogens from citric acid and l-lysine
Source: RSC Adv. 2026 Jan 13;16(4):2973–80. doi: 10.1039/d5ra09240j (PMC12797206; doi:10.1039/d5ra09240j)
Supplement: RA-016-D5RA09240J-s001 [file RA-016-D5RA09240J-s001.pdf]

Supplementary Information (SI) for RSC Advances.
This journal is © The Royal Society of Chemistry 2026

Supporting Information

Molecular Mechanism of Biocompatible Clusteroluminogens from Citric Acid and L-Lysine

Qiannan Zhang [1], Pingchuan Sun [2]*, Baohui Li [1]*

1. *School of Physics, Nankai University, Tianjin 300071, China*
2. *Key Laboratory of Functional Polymer Materials of Ministry of Education and College of Chemistry, Nankai University, Tianjin 300071, China*

*To whom correspondence should be addressed: spclbh@nankai.edu.cn; baohui@nankai.edu.cn

Table of Contents

1. FT-IR results 3
2. Low-field NMR Study Results Based on Grinding/Mixing of Citric Acid and Lysine at a 1:1 Molar Ratio 3
3. Theoretical Calculations 4
4. ^{1}H-NMR spectral of mild condition and physical mixture with a CA: Lys molar ratio of 1:1 in D_2O 6
5. Reproducibility, Repeatability, and Sensing Properties of CA/Lys Blends 7

1. FT-IR results

The FT-IR spectra (Figure S1a) reveal distinct absorption peaks at 2937 cm^{-1} and 2861 cm^{-1} in lysine, corresponding to the stretching vibrations of N-H···O and C=O···H, respectively. These peaks are nearly absent in pure citric acid (CA). Upon blending the two components, these characteristic peaks undergo notable modifications, suggesting that the incorporation of CA disrupts the original hydrogen-bonding network. For citric acid, the prominent peak at 1701 cm^{-1} is assigned to the C=O stretching vibration. With the addition of lysine, both the position and intensity of this peak exhibit changes. Specifically, a higher molar ratio of CA results in increased intensity at 1701 cm^{-1}. Concurrently, the characteristic peaks of lysine—the NH_3^+ bending vibration at 1571 cm^{-1} and the NH_2 deformation mode at 1504 cm^{-1}—also shift, indicating that blending alters the local chemical environment without fundamentally changing the molecular structure. Theoretical FT-IR simulations (Figure S1b) further support these observations. The simulated spectrum shows close alignment with experimental results, particularly for the C=O stretching vibration of CA (observed at 1852 cm^{-1}) and the NH_3^+ vibration of lysine (observed at 1731 cm^{-1}) in the blended system. This consistency between experimental and computational data allows for reliable structural interpretation of the hybrid material.

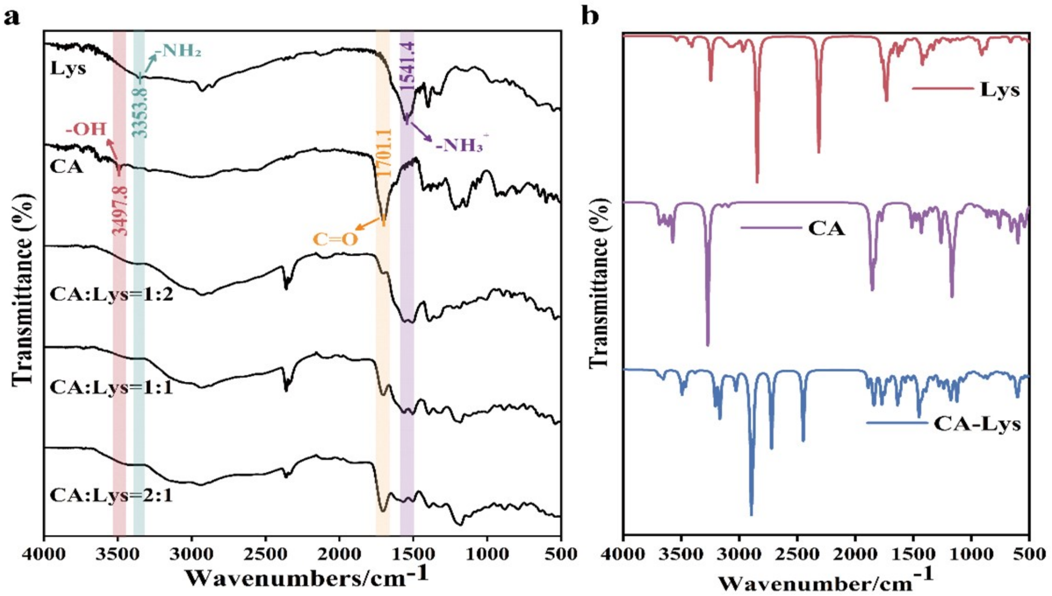

Figure S1. (a) Comparative experimental FT-IR spectra of pure citric acid (CA), pure lysine (Lys), and their binary mixtures at CA-to-Lys molar ratios of 2:1, 1:1, and 1:2. (b) Theoretical FT-IR spectra simulated for pure CA, pure Lys, and their equimolar (1:1) mixture.

2. Low-field NMR Study Results Based on Grinding/Mixing of Citric Acid and Lysine at a 1:1 Molar Ratio

Table S1. Low-Field NMR Study of Citric Acid–Lysine (1:1 Molar Ratio) Grinding/Mixing

	f^{Rigid}/%	T_2^{Rigid}/ms	f^{Mobile} /%	T_2^{Mobile} /ms	R2
Homogeneous grinding at a CA: Lys=1:1	95.75	0.01468	4.25	0.1	0.99925
Mixing of CA: Lys=1:1	63.31	0.01378	36.69	0.1	0.99978

Rigid represents the fraction of the rigid phase component, and Mobile stands for the fraction of the

flexible phase component.

3. Theoretical Calculations

All molecular structures were optimized using the Gaussian and Multiwfn programs with the density functional theory (DFT) method, employing the B3LYP functional and the 6-31G(d) basis set. Grimme's DFT-D3 correction was applied to better describe London dispersion effects and long-range intermolecular/intramolecular charge transfer processes. Additionally, frequency analysis calculations were performed at the same theoretical level to confirm that the optimized structures corresponded to local minimum. Time-dependent density functional theory (TD-DFT) was also conducted at the same level of theory to compute the optimized singlet geometries and energy levels. Polymer-related calculations were approximated using oligomers with four repeating units.

The three complexes exhibited nearly identical fluorescence emission wavelengths, their photophysical properties differed significantly from the pristine materials. Accordingly, the analysis focused on comparing each complex against its corresponding raw materials rather than conducting inter-comparisons among the different complex ratios. DFT calculations for the three CA: Lys ratios (2:1, 1:1, 1:2) reveal progressive changes in electronic structure (Figure S2). The energy gap (ΔE) systematically decreases from 5.29 eV (2:1) → 5.37 eV (1:1) → 5.89 eV (1:2). This narrowing bandgap correlates with enhanced long-wavelength fluorescence efficiency and explains the ratio-dependent photophysical evolution observed experimentally.

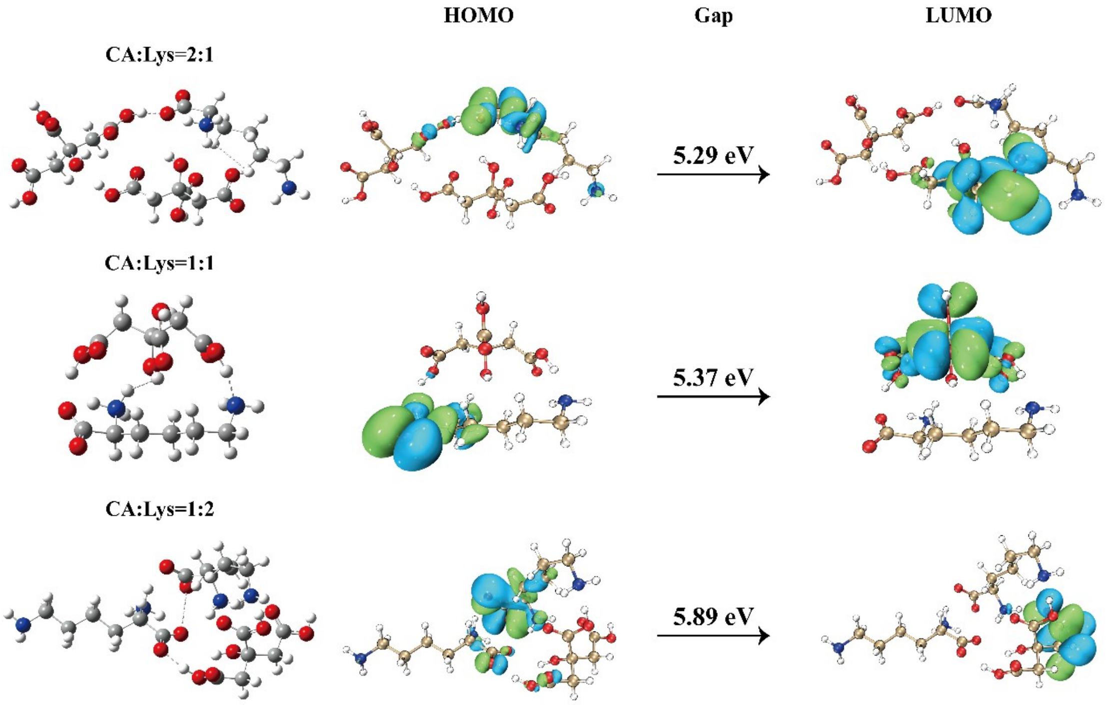

Figure S2. Frontier molecular orbital characteristics of optimized structures: CA: Lys=1:1, CA: Lys=1:2, and CA: Lys=2:1 hybrid system, illustrating HOMO-LUMO distributions and their contributions to fluorescence emission.

To strengthen the theoretical foundation of this study, we performed additional calculations on excited states of different configurations with identical composition. The theoretical calculation results reveal that CA-Lys blends with identical molar ratios exhibit distinct structural configurations, leading to varied emission wavelengths (Figure S3). This structure-dependent emission behavior may associate with the excitation-dependent effect (EDE) phenomenon.

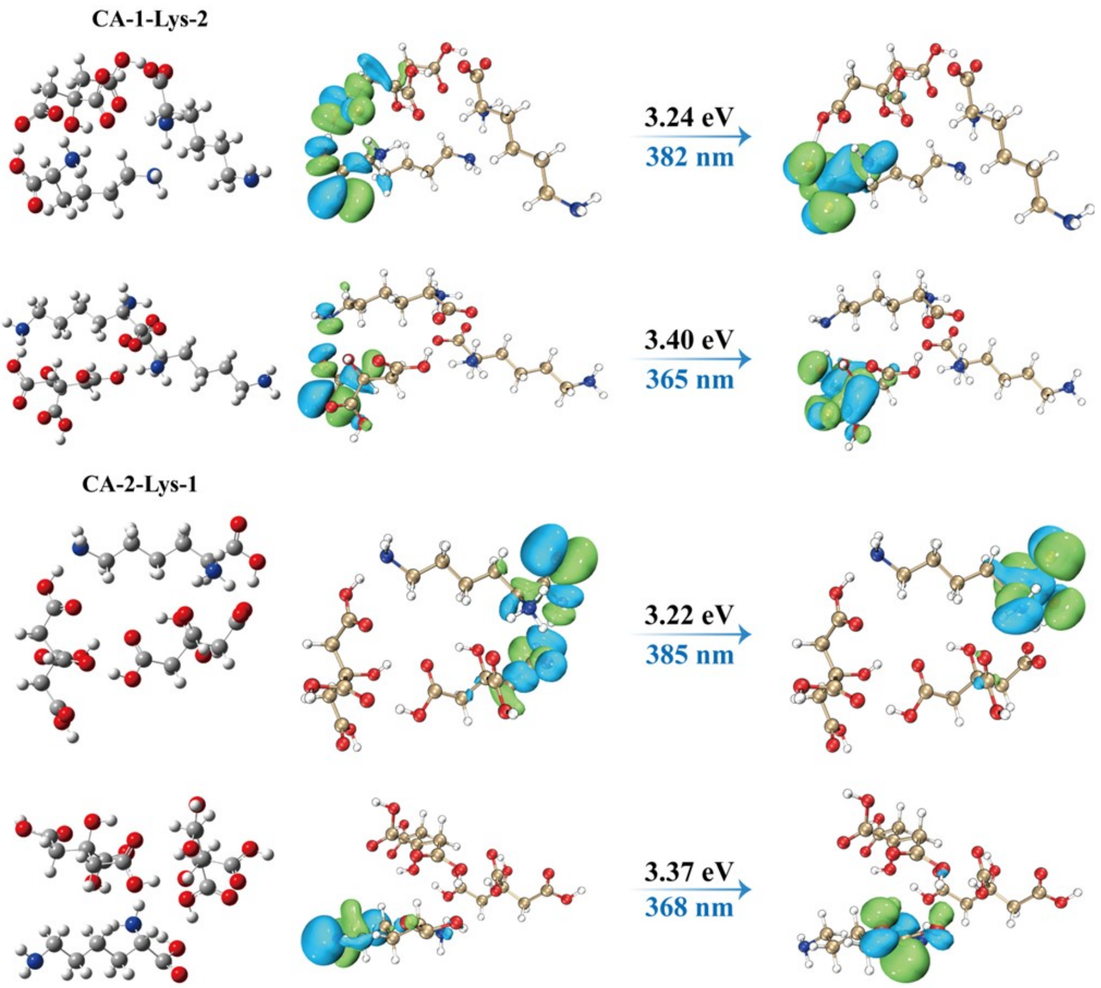

Figure S3. DFT-calculated electronic energy levels of S_1 for the CA: Lys=1:2 and CA: Lys=2:1 complex with different configurations.

As shown in the Figure S4, the DFT calculations results indicate that the energy level difference varies (HOMO-LUMO gap) with the lysine (Lys) content, exhibiting a decreasing trend as the Lys proportion increases. This phenomenon may be attributed to excitation-dependent effect.

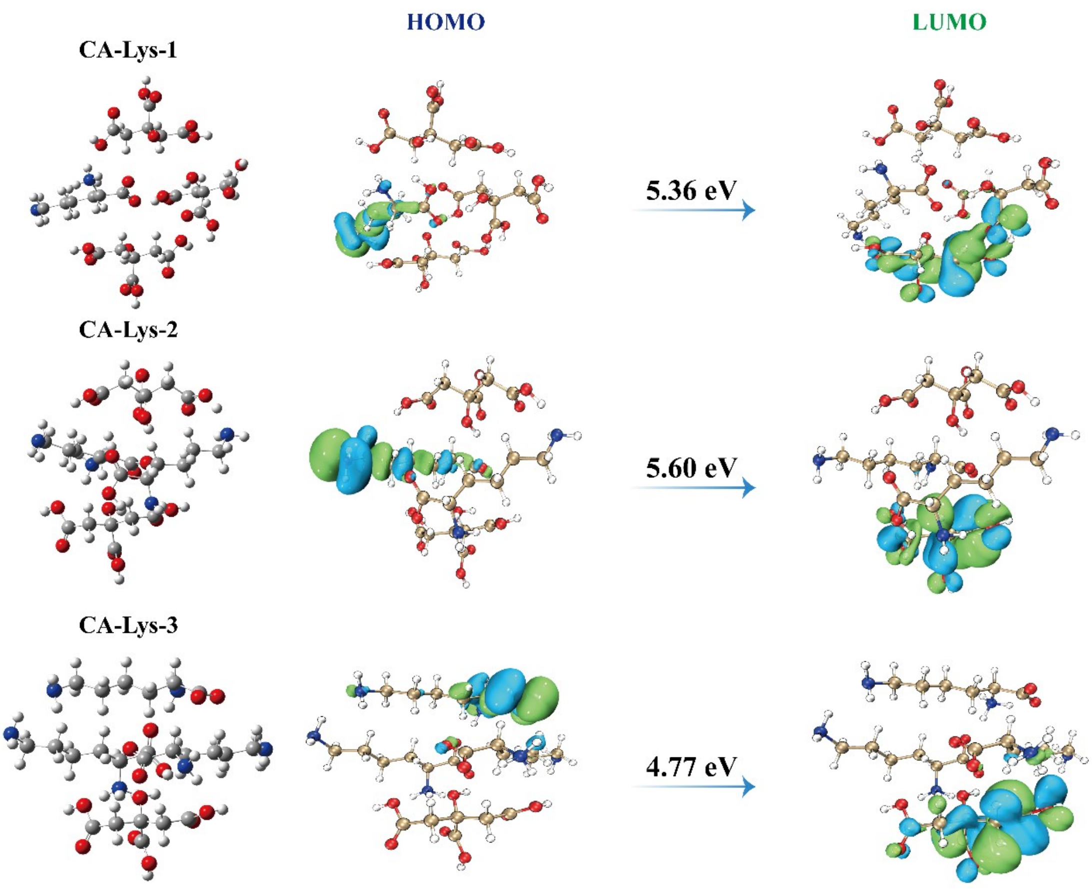

Figure S4. Frontier molecular orbital characteristics of optimized structures: hybrid system with Lys contents of 1, 2, and 3, illustrating HOMO-LUMO distributions and their contributions to fluorescence emission.

4. ^{1}H-NMR spectral of mild condition and physical mixture with a CA: Lys molar ratio of 1:1 in D_2O

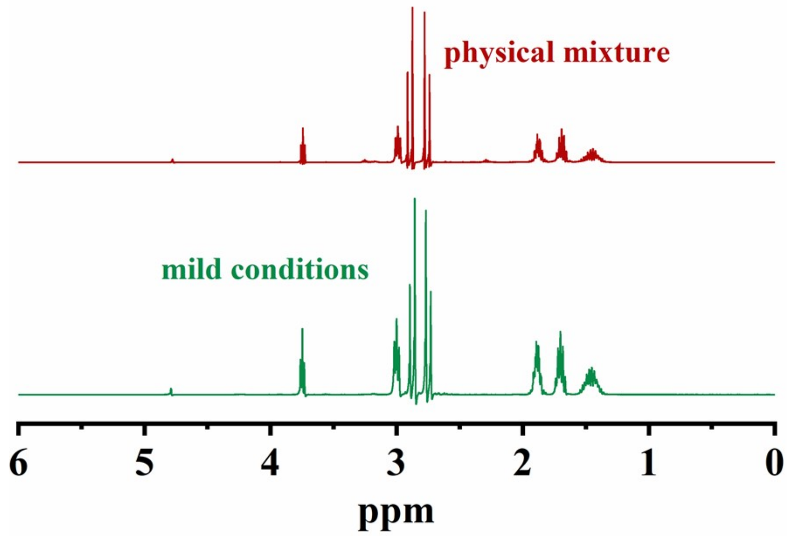

Figure S5. ^{1}H-NMR spectral of mild condition and physical mixture with a CA: Lys molar ratio of 1:1 in D_2O

As shown in Figure R1(a), the ^{1}H NMR spectra of the sample heated at 70°C and the physically

blended sample at room temperature were completely identical. This indicates that under our experimental conditions, no chemical reaction occurred between citric acid and lysine, further ruling out the possibility of carbon dot formation.

5. Reproducibility, Repeatability, and Sensing Properties of CA/Lys Blends

To test the sustainability of their material through examining repeatability, we fabricated new composite films by blending CA-Lys complexes with PVA. As shown in Figure S6a-c, the fluorescence spectra and quantum yield measurements of these films are in good agreement with our previously reported data, with minor intensity variations that may arise from inherent instrument error in the fluorometer. Figure S6d displays the photoluminescence spectrum of a recast CA-Lys-embedded PVA composite film after dissolution and re-deposition, which also aligns well with our earlier findings. These results confirm the excellent reproducibility, repeatability, and sensing performance of our material system.

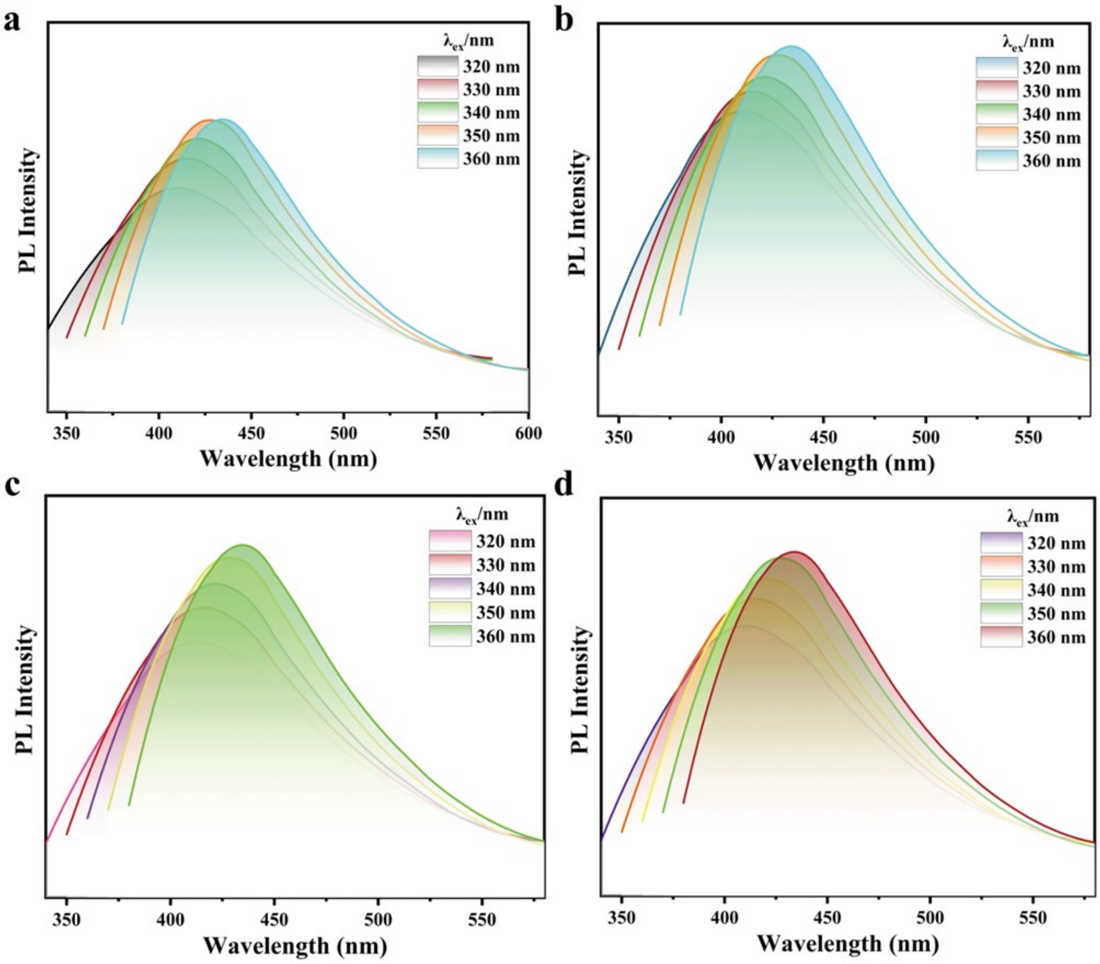

Figure S6. Photoluminescence (PL) spectra of CA-Lys-embedded PVA composite film(a-c) prepared by three separate fabrication and recast CA-Lys-embedded PVA composite film after dissolution(d).

Theoretical Molecular Structure of CA-Lys in Fig. 6a-d

C	0.52761181	-1.10291144	-1.09949469
C	-1.69124415	-3.41687756	0.92912713
C	1.71153677	-0.92913878	-0.19704297

C	-2.42695660	-3.23043176	-0.38622189
C	2.82808645	-1.96754628	-0.40719172
C	-3.26605658	-1.93247565	-0.36427247
C	3.95985660	-1.66658241	0.59975330
C	-3.96489986	-1.73867552	-1.72935501
C	5.17622357	-2.53308636	0.35805863
C	-4.74233662	-0.43427234	-1.71274921
C	3.35111313	-1.86944977	-1.85681835
C	-4.32237833	-1.98787094	0.76085564
H	1.30956112	-1.00673440	0.81960643
H	-1.72135366	-3.19058392	-1.21604244
H	4.27684716	-0.62766731	0.49205678
H	-4.65850185	-2.56274653	-1.90886098
H	3.55958838	-1.81781917	1.60423237
H	-3.19596432	-1.72599308	-2.50639093
H	6.19706078	-3.91724513	1.10654670
H	-4.62077840	1.43093700	-2.09050718
H	4.07531780	-0.64175691	-3.08910723
H	-5.76240548	-3.03444580	1.35245169
H	1.61002829	-3.43536561	-0.70699664
H	-1.97471415	-0.82541172	0.70257963
O	0.21314940	-2.13242479	-1.66151811
O	-2.20248980	-3.29432542	2.01914755
O	5.41389221	-3.39541834	1.36664938
O	-4.10785858	0.57102317	-2.30798425
O	5.87623723	-2.45258705	-0.62976691
O	-5.82304639	-0.33863813	-1.14703120
O	3.65886327	-0.56649497	-2.20916187
O	-5.11556220	-3.07209526	0.62364447
O	3.45895177	-2.77486259	-2.63617431
O	-4.45411336	-1.16967419	1.63878085
O	2.39387696	-3.27646140	-0.14751202
O	-2.45125620	-0.79202168	-0.18657328
H	-3.08993649	-4.09107881	-0.52441067
H	2.11537297	0.07580839	-0.31272779
O	-0.20453365	0.03631066	-1.17890292
H	-1.17216865	-0.23199300	-1.20089766
O	-0.39065137	-3.73703755	0.75044493
H	0.02822037	-3.76857378	1.63364628
C	-1.36470277	1.65947633	1.95918028
H	-1.46879762	1.79213982	0.87762506
C	-2.74057967	1.48053174	2.59737786
H	-3.07844289	0.45955980	2.40266755
H	-2.63268849	1.57241417	3.68816466

C	-3.78537126	2.47380995	2.05104375
H	-3.36359314	3.49184523	2.01660681
H	-4.63849264	2.52250986	2.73938432
C	-4.27938735	2.05473412	0.65944501
H	-4.86594172	1.13608021	0.76381302
H	-3.43444310	1.79121076	0.01406365
C	-5.10687203	3.12201312	-0.05613686
H	-4.47913358	3.99297567	-0.28083542
H	-5.92324716	3.47247852	0.59592911
N	-0.66351150	2.88841459	2.48198513
C	-0.36679314	0.47981108	2.15722617
O	0.78680610	0.79990484	2.55154748
O	-0.79138059	-0.65883478	1.85573352
H	0.22669733	2.48133482	2.85536127
H	-0.41745680	3.59733364	1.70399430
N	-5.62494413	2.58494419	-1.33692493
C	1.58550467	2.81300655	-0.96939682
C	2.92400565	3.36095865	-0.47868671
H	3.43971595	3.86271450	-1.30998908
H	2.68441649	4.14585247	0.24845707
C	3.82577230	2.30668664	0.17663313
H	4.05768232	1.50862365	-0.54433761
H	3.26883089	1.83745121	0.99915481
C	5.14471083	2.88169913	0.70151052
H	4.95181123	3.66719170	1.44250001
H	5.69055703	3.36125223	-0.12539689
C	6.03474364	1.81276532	1.34113755
H	6.20627841	0.99942574	0.60860561
H	5.49857094	1.36171602	2.18659257
C	0.66216094	3.95430587	-1.53053067
O	0.01831089	4.63405909	-0.69006847
O	0.70722237	4.08108881	-2.77181611
N	7.26605221	2.41464240	1.86267114
N	1.74461739	1.90940347	-2.16735449
H	1.59285106	2.64343560	-2.91335233
H	1.10525844	2.23943832	-0.17698829
H	0.93628688	1.26618851	-2.17915165
H	-6.07793372	3.31834096	-1.87990624
H	-6.32489688	1.86686686	-1.14145129
H	7.84228194	2.73716077	1.08577328
H	7.81886887	1.70902558	2.34663945
H	-1.19164309	3.35498374	3.22051152
H	2.61109958	1.36432541	-2.21847104
